# DCs Pulsed with Novel HLA-A2-Restricted CTL Epitopes against Hepatitis C Virus Induced a Broadly Reactive Anti-HCV-Specific T Lymphocyte Response

**DOI:** 10.1371/journal.pone.0038390

**Published:** 2012-06-12

**Authors:** Zhongsheng Guo, Henghui Zhang, Huiying Rao, Dong Jiang, Xu Cong, Bo Feng, Jianghua Wang, Lai Wei, Hongsong Chen

**Affiliations:** 1 Hepatology Institute, Peking University, Peking University People’s Hospital, Beijing, China; 2 Beijing Key Laboratory of Hepatitis C and Immunotherapy for Liver Diseases, Beijing, China; Fudan University, China

## Abstract

**Objective:**

To determine the capacity of dendritic cells (DCs) loaded with single or multiple-peptide mixtures of novel hepatitis C virus (HCV) epitopes to stimulate HCV-specific cytotoxic T lymphocyte (CTL) effector functions.

**Methods:**

A bioinformatics approach was used to predict HLA-A2-restricted HCV-specific CTL epitopes, and the predicted peptides identified from this screen were synthesized. Subsequent IFN-γ ELISPOT analysis detected the stimulating function of these peptides in peripheral blood mononuclear cells (PBMCs) from both chronic and self-limited HCV infected subjects (subjects exhibiting spontaneous HCV clearance). Mature DCs, derived in vitro from CD14^+^ monocytes harvested from the study subjects by incubation with appropriate cytokine cocktails, were loaded with novel peptide or epitope peptide mixtures and co-cultured with autologous T lymphocytes. Granzyme B (GrB) and IFN-γ ELISPOT analysis was used to test for epitope-specific CTL responses. T-cell-derived cytokines contained in the co-cultured supernatant were detected by flow cytometry.

**Results:**

We identified 7 novel HLA-A2-restricted HCV-specific CTL epitopes that increased the frequency of IFN-γ-producing T cells compared to other epitopes, as assayed by measuring spot forming cells (SFCs). Two epitopes had the strongest stimulating capability in the self-limited subjects, one found in the E2 and one in the NS2 region of HCV; five epitopes had a strong stimulating capacity in both chronic and self-limited HCV infection, but were stronger in the self-limited subjects. They were distributed in E2, NS2, NS3, NS4, and NS5 regions of HCV, respectively. We also found that mDCs loaded with novel peptide mixtures could significantly increase GrB and IFN-γ SFCs as compared to single peptides, especially in chronic HCV infection subjects. Additionally, we found that DCs pulsed with multiple epitope peptide mixtures induced a Th1-biased immune response.

**Conclusions:**

Seven novel and strongly stimulating HLA-A2-restricted HCV-specific CTL epitopes were identified. Furthermore, DCs loaded with multiple-epitope peptide mixtures induced epitope-specific CTLs responses.

## Introduction

Chronic hepatitis C virus (HCV) infection is a serious health problem worldwide. There are estimated to be more than 170 million carriers of hepatitis C infection. Approximately 3 or 4 million new cases arise every year [1], [2]. Moreover, chronic HCV infection is a potential cause of end-liver disease, such as liver cirrhosis, hepatocellular carcinoma, and hepatic failure [3]. Currently, the standard therapy is a combination therapy of pegylated interferon (Peg-interferon) and ribavirin. However, the treatment efficiency of this therapy is variable depending on the HCV genotype; it is approximately 50% effective in treating HCV genotypes 1 and 4 and approximately 80% effective in treating HCV genotypes 2 and 3. In addition, combination therapy has severe adverse side effects as well as a high cost [4], [5]. Moreover, the high mutation rate of HCV and lack of effective animal models have restricted the development of preventive and therapeutic vaccines [6]. Therefore, exploration of new therapeutic strategies is urgently needed.

The cellular immune response in both the innate and adaptive immune responses is significantly impaired during chronic HCV infection. Dendritic cells (DCs) are professional antigen presentation cells (APCs) that play a crucial role in innate immunity and in priming T lymphocytes for the adaptive immune response. However, previous studies have shown that DC function is impaired in persistent HCV infection, which results in ineffective T cell priming [7]–[10]. Consistent with this, previous reports have also shown that while there is a strong multi-specific or multi-epitope CD8^+^ or CD4^+^ T lymphocyte response in self-limited HCV infection, there is a narrow and weak T cell response in persistent HCV infection [11], [12]. In addition, other studies found that T cells from chronic HCV infected subjects exhibited high levels of PD-1 or Tim3 [13], [14]; these surface markers correlated with an anergic or exhausted state.

Because T cells play a vital role in determining whether HCV infection persists or is eliminated from the host, one promising strategy is to induce or enhance the T lymphocyte response. A numbers of studies have shown that peptide-specific cytotoxic T lymphocytes (CTLs) could be induced using recombinant DNA or a protein vaccine *in vitro*; some of these strategies were even developed into clinical trials to treat chronic HCV subjects [15]–[17]. However, the antiviral effect of these induced peptide-specific CTLs is limited. Another developed therapeutic strategy is to use DCs loaded with corresponding antigen in tumor subjects that can induce a peptide-specific CTL response [18]–[20]. Last year, the DC vaccine PROVENG was approved by the FDA to treat prostate cancer [21]. DC immunotherapy has even been used to treat HCV-infected individuals in a phase I clinical trial, where DCs were loaded with lipopeptide; this treatment was determined to be safe and was well tolerated [22]. Based on this encouraging clinical result, DC-based immunotherapy to treat chronic hepatitis C is expected to be an emerging field of research [23], [24].

In this study, we used bioinformatics to predict HLA-A2-restricted HCV-specific CTL epitope peptides and subsequently evaluated their potential to stimulate PBMCs using IFN-γ ELISPOT as a readout. We then tested the T-lymphocyte stimulating capacity of DCs pulsed with selected immunodominant single peptides or multiple-epitope peptide mixtures to determine whether this would be an efficient strategy to induce a more effective T cell response against HCV that would eventually clear persistent infection. We found that these novel peptides could induce a Th1-biased T cell immune response, suggesting that these peptides could improve the frequency of effective anti-HCV T cells that could eventually eliminate the HCV infection from chronically infected hosts.

## Materials and Methods

### Study Subjects

The study population consisted of 6 HLA-A2-positive chronic hepatitis C (CHC) infected subjects and 5 HLA-A2–positive HCV subjects that demonstrated spontaneous HCV clearance (hereafter called self-limited). We categorized CHC subjects as being positive for anti-HCV Abs as well as being HCV RNA positive, while self-limited HCV subjects were positive for anti-HCV Abs but were HCV RNA negative. The subjects were infected with hepatitis C by apheresis plasma reinfusion in the 1990s. CHC subjects were not treated during the six months before this study. Study subjects also positive for other conditions, including HBV, HIV, autoimmune hepatitis, and serious heart or lung disease, were excluded from the study. The detailed characteristics are listed in Table 1. Written informed consent was obtained from all subjects. The protocol of the study was approved by the ethics committee of Peking University People’s Hospital.

**Table 1:** Demographic characteristics of the subjects.

State	No	Gender	Age (yr)	HCV^#^	HCV RNA?	ALT*	Co-existing disease
**self-limited infection**	1	F	45	15.15	negative	12	no
	2	M	59	8.71	negative	15	no
	3	M	60	5.83	negative	18	fatty liver
	4	M	58	6.46	negative	12	fatty liver
	5	F	43	6.17	negative	13	no
**chronic infection**	6	F	45	14.76	4.91E+06	18	no
	7	M	50	15.19	4.88E+06	20	no
	8	M	39	12.75	1.31E+07	44	alcohol
	9	F	41	12.45	9.74E+05	26	no
	10	M	47	14.65	2.02E+04	134	no
	11	M	50	11.29	1.26E+07	31	fatty liver

Note: ^#^HCV Abs detected by ELISA (S/Co, Abbott).

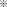
Virus loads were quantified through quantitative real-time Taqman PCR (IU/mL, Roche).

* ALT (IU/mL);

“no” means no combined diseases.

### Epitope Peptides Prediction and Synthesis

For peptide prediction, we chose an HCV prototype (genotype 1b) isolated in China from Genbank (L02836.1) [25]. The online program CTLPred was used to predict HCV-specific CD8^+^ T-lymphocyte epitopes [26]. The criteria for identifying candidate epitope peptide were as follows: (1) HLA-A2 restricted; (2) relatively conserved in HCV genotypes 1 and 2; (3) high epitope score compared to the threshold (Cut off = 0.36, which has a high sensitivity, specificity, precision and accuracy); (4) unique sequence has not been previously reported. Moreover, we applied text mining by using keywords in Pubmed and selected several HLA-A2-restricted hepatitis C-specific CTL epitopes, which were reported to have a stronger stimulating capability of IFN-γ. And we want to know whether these epitope peptide had the same stimulatory function in the Chinese.

The 9-mer peptides were synthesized using an automated multiple-peptide synthesizer (Invitrogen, Shanghai, China) and the purity was assessed to be >98% through high-performance liquid chromatography (HPLC) testing. The molecular mass is consistent with the predicted value by mass spectrography analysis. All peptides were solved in DMSO (Sigma, Shanghai, China), diluted in sterile PBS, and stored at −80°C for subsequent use.

### Screening and Identifying Epitope Peptides by IFN-γ ELISPOT

IFN-γ ELISPOT (BD Pharmingen, San Diego, CA, USA) was used to functionally screen epitope peptides. The protocol used in this study was previously described [27], [28] and manufacturer’s recommended protocol was followed. Briefly, polyvinylidene plates (96-well, Millipore, Billerica, MA, USA) were coated with 2.5 µg/mL recombinant anti-human IFN-γ Abs (BD, Pharmingen) in PBS at 4°C overnight and washed one time with complete RPMI (cRPMI) 1640 medium (Gibco/Invitrogen, Carlsbad, CA, USA) containing 10% FBS (Gibco/invitrogen, Mulgrave Vic, Australia). After blocking with cRPMI 1640 for 2 h at room temperature, fresh or previously frozen PBMCs were added at 2×10^5^ cells/well in 100 µL of cRPMI 1640 media. Then, the synthesized peptides were directly added to the well at a final concentration of 10 µg/mL. The plates were incubated for 20 h at 37°C in 5% CO_2_. The cells were removed and the plates were washed 2 times with distilled water and subsequently washed 3 times with PBS containing 0.05% Tween-20 (PBS-T) (Sigma, Shanghai, China). The plates were developed with biotin-labeled anti-IFN-γ for 2 h at room temperature and then washed 3 times with PBS-T. The plates were then incubated with streptavidin-HRP for 1 h at room temperature, washed 4 times with PBS-T, and washed 3 times by PBS. To develop the plate, 3-Amino-9-ethylcarbazole (AEC) substrate (BD Pharmingen, San Diego, CA, USA) was added, and the color was allowed to develop for approximately 10 min. The reaction was stopped by distilled water and dried by air at room temperature. The spots were counted using an ELISPOT reader (Cellular Technology Ltd, USA) and analyzed by the Immunospot 5.0.3 Professional (Cellular Technology Ltd, USA) software. Each epitope was run in triplicate and the data was expressed as the number of spot-forming cells (SFCs) per 2×10^5^ PBMCs. Phytohemagglutinin (PHA, Sigma, Shanghai, China) served as a positive control for T cell stimulation. An HLA-A2–restricted CTL epitope (WLSLLVPFV) from an envelope protein from the hepatitis B virus (HBV) was selected as an unrelated epitope peptide control. PBMCs cultured in medium without any peptides were used as a negative control. If the SFC per tested well at least 25 SFCs/2×10^5^ PBMCs after the SFC value of the negative control was subtracted from the raw number and the stimulatory function in self-limited HCV infection was stronger than in the chronic infection, the response was considered positive.

### Isolation of PBMCs and Monocyte-derived DC Generation

PBMCs from chronic HCV infection and self-limited HCV infection subjects were isolated by density gradient centrifugation using Ficoll-Paque Plus (GE Health Bio-science, AB, Sweden) as described [29]. Untouched monocytes from fresh or cryopreserved PBMCs were separated by negative selection by the monocyte isolation Kit II (Miltenyi Biotec, Bergisch Gladbach, Germany). The magnetically labeled monocyte-depleted cells were frozen in liquid nitrogen as the source of autologous T lymphocytes. The purity of CD14^+^ monocytes by flow cytometry was >90% and the viability of monocytes was >95% by trypan blue staining.

The DC generation procedure was followed as previously described by Romani et al. and Kim et al. [30], [31] with slight modifications. Briefly, monocytes (2×10^6^/well) were incubated for 5 days in AIM V medium (Gibco/Invitrogen, Carlsbad, CA) containing 800 U/mL of animal-free (AF) recombinant interleukin 4 (rhIL-4) (Peprotech, Rocky Hill, NJ, USA) and 1000 U/mL AF-recombinant granulocyte-monocyte colony-stimulating factor (rhGM-CSF) (Peprotech, Rocky Hill, NJ, USA) to induce immature DC (iDC). Fresh medium containing these cytokines was replaced every 3 days. On day 5, to induce mature DCs (mDCs), iDCs were exposed to a cytokine cocktail containing AF-recombinant IL-1β (1000 U/mL) (Peprotech, Rocky Hill, NJ, USA), IL-6 (1000 U/mL) (Peprotech, Rocky Hill, NJ, USA) and TNF-α (1000 U/mL) (Peprotech, Rocky Hill, NJ, USA). After 6 h, a 10 µg/mL final concentration of PolyI:C (Invivogen, San Diego, CA, USA) was added to iDCs and cultured for 48 h. At day 7 or 8, the DC culture supernatant was collected and frozen at −80°C. Then, mDCs were harvested and stained with PE or FITC-conjugated antibodies against CD11c, CD80, CD83, CD86, CD14, and HLA-DR and their appropriate isotype-matched regents (BD Pharmingen, USA). Cell surface expression was assayed by flow cytometry (BD FACSCalibur, San Jose, CA), and Cellquest was used to analyze the magnitude of surface molecule expression on the mDCs. IL-12 and IL-10 levels were determined by enzyme-linked immunosorbent assay (ELISA) (Bender MedSystems, Vienna, Austria) according to manufacturer’s instructions.

### Induction of Cellular Immune Response and IFN-γ/GrB ELISPOT Assay

mDCs were harvested and incubated with the DNA crosslinker mitomycin C (30 µg/mL, Roche, Shanghai) to inhibit DC proliferation and cytokine production for 40 min at 37°C in 5% CO_2_. mDCs were then washed 4 times with PBS and pulsed with selected single peptide or multiple-epitope peptide mixtures for 2 h at at 37°C in 5% CO_2_ in 96-well round plates. The autologous cryopreserved PBMCs were used as the source of responder T lymphocytes in the stimulation experiments. The autologous responder T cells (2×10^5^/well) were cocultured with mDCs loaded with single peptide or multiple-epitope peptide mixtures as 10∶1 ratio for 72 h. Each peptide was added at a final concentration of 10 µg/mL. The next day, AF-recombinant human IL-2 (30 U/mL) (Peprotech, Rocky Hill, NJ, USA) was added to the co-culture system. Single peptide or multiple-epitope peptide mixtures were added into triplicate wells. T cell responder cells were harvested and transferred to the pre-coated IFN-γ or Granzyme B (GrB) ELISPOT plates (BD Pharmingen, San Diego, CA). The corresponding single peptide or multiple-epitope peptide mixtures were added again to the ELISPOT wells and incubated for 20 h at 37°C in 5% CO_2_. The ELISPOT plates were developed as described above.

### Cytokine Detection in Co-culture Supernatant

On day 4, the supernatant from DC co-culture with autologous responder T lymphocytes was collected and fast frozen at −80°C before the responder T cells were transferred to the ELISPOT plates. The frozen supernatants were subsequently thawed, and IL-2, IFN-γ, IL-4, and IL-10(Human Th1/Th2 11plex FlowCytomix Multiplex, Bender MedSystems/eBioscience, Vienna, Austria) were detected by flow cytometry at the same time according to the manufacturer’s instructions and quantified using FlowCytomix Pro 2.4 Software (eBioscience, USA).

### Statistical Analysis

The continuous variables were expressed as mean±SEM. The student’s *t*-test and one-way *ANOWA* were used to assess the statistical difference between the different stimulatory groups. The data was analyzed through SPSS V13.0 (SPSS Inc. Chicago, IL, USA). A value of *P*<0.05 was considered significant.

## Results

### Seven Novel HLA-A2 Restricted HCV-specific CTL Epitopes were Identified by Bioinformatics Screening and Functioned to Induce IFN-γ Production from PBMCs

**Figure 1:**
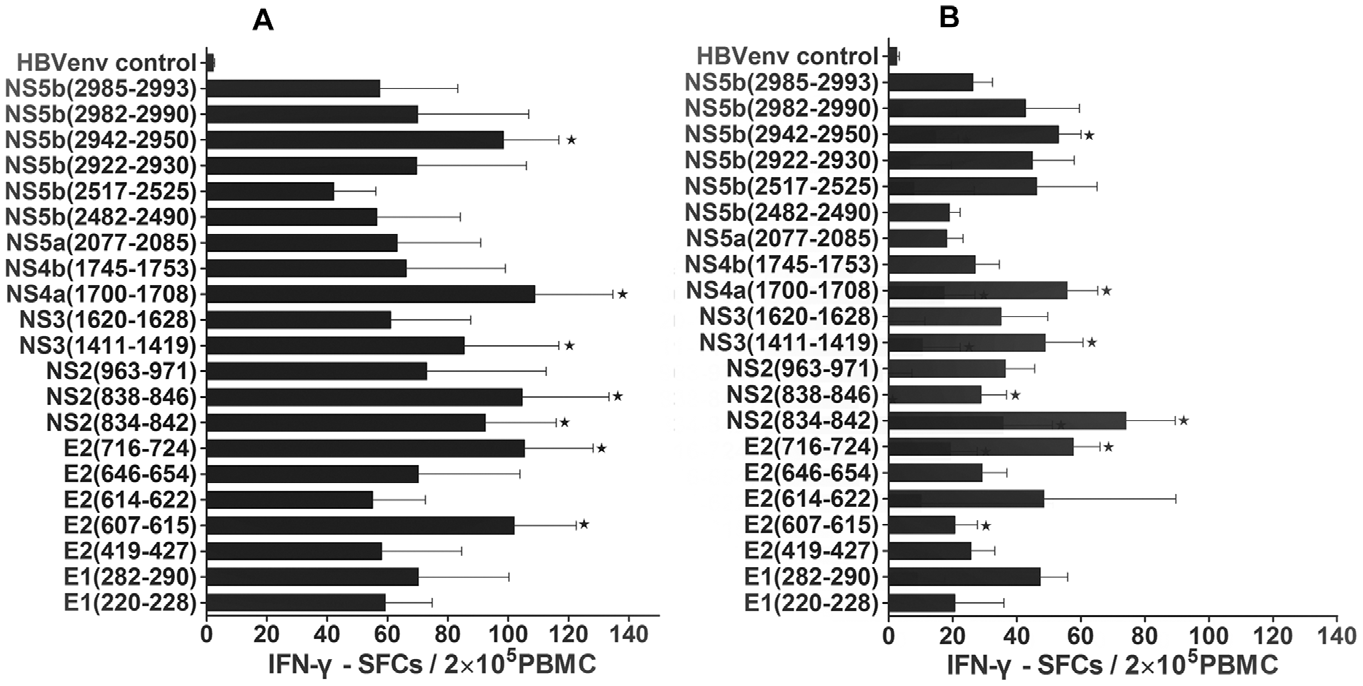
IFN-γ SFCs of all predicted epitope peptides in study subjects. The data was expressed as mean±SEM and IFN-γ SFCs per epitope peptide, with the SFCs values from the negative control subtracted. The data from 5 self-limited and 6 chronic HCV infection subjects was included. Triplicate wells of every peptide in the same experiments were used. **A**. IFN-γ SFCs from the predicted peptides in self-limited HCV infection. **B**. IFN-γ SFCs from the predicted peptides in chronic HCV infection. “**^★^**” stands for positive epitope peptide, which had the strongest stimulatory function of IFN-γ secretion in self-limited HCV infection and defined by >25 SFCs/2×10^5^ PBMCs after subtracting the negative control SFCs value.

To identify previously unknown HLA-A2-restricted epitopes in HCV that are likely to prime CD8^+^ T cells when presented by DCs, a bioinformatics approach was used to identify suitable candidates. Based on bioinformatics analysis using the criteria set forth in the *Materials and Methods*, 21 HCV HLA-A2-restricted epitopes were identified as candidates for CTL recognition; they were identified by having higher scores than the threshold, which we set at 0.36. Then we synthesized these 21 peptides and functionally tested them by choosing which peptides could induce peptide-specific IFN-γ secretion from CTLs in PBMCs harvested from both self-limited and chronic HCV subjects by IFN-γ ELISPOT assay. Using this assay, 7 functional peptides were selected for their simulation ability (Figure 1 and Table 2) according to our positive criteria in the *materials and methods* section. These seven peptides were found to be located in all HCV regions.

**Table 2:** Hepatitis C virus CTL epitopes prediction and identification.

Epitopes	Position	Sequence	The number of positive subjects	
			Self-limited(n)	Chronic infection (n)
CTL-1^△^	E2 (607–615)	CLVDYPYRL	2/5^*^	0
CTL-2^△^	NS2 (838–846)	FLARLIWWL	2/5	0
CTL-3^△^	E2 (716–724)	WEYVLLLFL	2/5	3/6
CTL-4^△^	NS2 (834–842)	YYKVFLARL	2/5	2/6
CTL-5^△^	NS3 (1411–1419)	GLNAVAYYR	2/5	2/6
CTL-6^△^	NS4a (1700–1708)	VLYEAFDEM	2/5	2/6
CTL-7^△^	NS5b (2942–2950)	KYLFNWAVK	3/5	2/6
CTL-8♦	E2 (723–731)	FLLLADARV	2/5	0
CTL-9♦	NS3 (1073–1081)	CINGVCWTV	3/5	3/5
CTL-10♦	NS3 (1585–1593)	YLVAYQATV	1/5	0
CTL-11♦	NS5b (2594–2962)	ALYDVVTKL	3/5	3/5

Note:^△^ stands for novel predicted epitopes.

♦ stands for past reported epitopes.

★ means two positives in five subjects.

Comparing the PBMC response to each peptide between the self-limited and chronic HCV infection, we observed some differences in stimulation capability. Only 2 of the 7 peptides had the strongest stimulating capability in the self-limited HCV infection PBMCs alone according to our criteria; these peptides originated from the E2 (607–615) and NS2 (838–846) region. Five peptides had a strong stimulating ability in both self-limited and chronic HCV patient PBMCs. However, the peptide-induced stimulation in the self-limited HCV infection was stronger than in the chronic HCV infection. These 5 peptides corresponded to the following HCV proteins: E2 (716–724), NS2 (834–842), NS3 (1411–1419), NS4a (1700–1708), and NS5b (2942–2950). We subsequently used these selected peptides in the following experiments to study the stimulation capability of DCs between the self-limited and chronic HCV infections.

Taken together, we identified 7 novel HCV epitope peptides that stimulated IFN-γ secretion by bioinformatics screening and subsequent functional studies in PBMCs from patient samples. In addition to these novel HCV peptides, our screen also identified nine previously reported HLA-A2-restricted HCV-specific CTL epitopes by text mining; of these, four peptides were selected as positive according to our criteria for stimulating IFN-γ production by ELISPOT assay. The detailed information about all peptides identified in this study is listed in Table 2.

### Activation Expression Markers and Cytokine Production from Monocyte-derived DCs Induced to Mature *in vitro* were not different between Self-limited and Chronic HCV Infected Subjects

In order to obtain mature DCs from self-limited and chronic HCV infected subjects to test the CTL-stimulation capacity of the novel peptides we identified, we harvested CD14^+^ monocytes from PBMCs and induced them to differentiate and develop into mDCs using the cytokine cocktail regimen detailed in the *Materials and Methods* section. To determine whether mDCs were produced, and to test whether there were any differences in activation status between the mDCs derived from self-limited or chronic HCV infected subjects, we first assessed the activation status by measuring activation marker expression by flow cytometry on day 8 of maturation. We found high levels of mDC-specific markers CD80, CD86, CD83, CD11c, and HLA-DR on all DCs; additionally, CD14 expression was downregulated by day 8. This expression data indicated that these cells differentiated from the harvested CD14^+^ monocytes into mDCs (Figure S1). Comparing the DCs, there was no difference in the mDC phenotype between DCs derived from self-limited and chronic HCV infection subjects (P>0.05). This result was consistent with some previous reports [27], [31]. In addition, we investigated the cytokine profile produced by the mDCs since the Th1-skewing cytokine IL-12 is associated with promoting CTL function. We found that the mDCs had higher levels of IL-12 than IL-10 (a cytokine associated with Th2-skewing). However, there was no significant difference between the mDCs derived from the self-limited HCV infection and chronic hepatitis C infection (data not shown). These data indicated that the mDCs derived from self-limited HCV infection and chronic hepatitis C infection are functionally similar, and that mDCs could induce a Th1 immune response during T cell priming.

### Mature DCs Pulsed with Multiple Epitope Peptide Mixtures were Able to Prime T Cell Responses

To evaluate the stimulation capability of selected peptides when presented by the mDCs, we tested stimulation by measuring IFN-γ production from responding T cells on a per cell basis by ELISPOT analysis. We first combined several peptides in different combinations, including 6 peptides identified earlier in the study and 3 previously reported peptides, to induce a strong and broad T cell response. Fourteen unique epitope peptide pools were created (Table S1); the peptides derived from both the structural and non-structural regions in HCV to prime T cells against diverse HCV targets. Confirming that PBMCs pulsed with these 14 peptide pools skewed toward Th1 rather than Th2 T cell differentiation, each pool induced more Th1 cytokine IFN-γ production than the Th2 cytokine IL-4 (data not shown).

**Figure 2:**
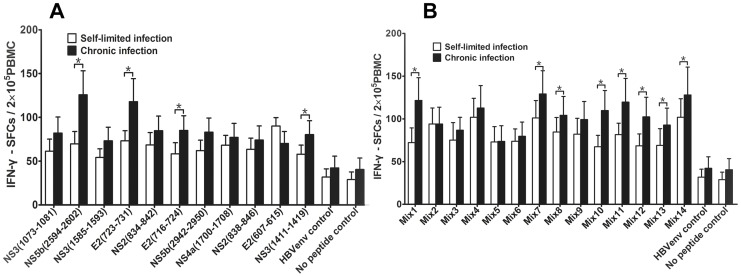
The frequency of IFN-γ-producing CTLs from PBMCs cocultured with mDC loaded with peptides. The data was expressed as mean±SEM. The data from 5 subjects for every group was included. Triplicate wells of every peptide in the same experiment were used. The single peptides include 4 previously reported epitope peptides (E2 (723–731), NS3 (1073–1081), NS3 (1585–1593), and NS5b (2594–2602)), 7 predicted peptides and HLA-A2-restricted CTL HBV envelope epitope peptide (WLSLLVPFV) as an unrelated peptide control. The epitope peptide mixtures (mix1∼mix14) contains 2 to 6 single epitope peptides in each mixture. **A**. IFN-γ SFCs induced by single peptide in self-limited and chronic HCV infection. **B**. IFN-γ SFCs induced by multiple epitope peptide mixtures in self-limited and chronic HCV infection; *P<0.05.

We found that DCs pulsed with either single or multiple-epitope peptide pools could induce significantly more IFN-γ SFCs in both self-limited HCV infection and chronic HCV infection than the HLA-A2-restricted CTL HBV envelope control and no peptide control (Figure 2A–B). And the number of IFN-γ SFCs was especially evident in chronic HCV infection-derived mDCs pulsed with the single peptides or the multiple-epitope peptide combinations (Figure 2).In addition, we found that several multiple-epitope peptide combinations could significantly induce IFN-γ SFCs in chronic HCV infection compared to self-limited HCV infection(P<0.05, Figure 2B).This result showed that the immunodominant epitopes of multiple- epitope peptide mixtures could stimulate significant levels of IFN-γ production from effector T cells and suggests that they could exert a protective immune response.

### Mature DCs Pulsed with Multiple Epitope-peptide Mixtures Induced Epitope-specific Cytotoxic Responses from CTLs

**Figure 3:**
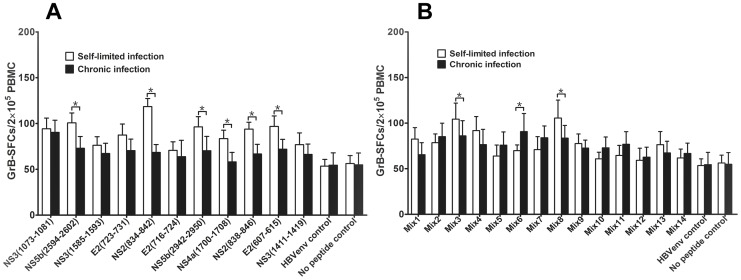
The frequency of GrB-producing CTLs from PBMCs cocultured with DC pulsed with epitope peptides. The data was expressed as mean±SEM. The data from 5 subjects from every group was included. Triplicate wells from every peptide in the same experiment were used. The single peptides include 4 previously reported epitope peptides (E2 (723–731), NS3 (1073–1081), NS3 (1585–1593), and NS5b (2594–2602)), 7 predicted peptides and HLA-A2 restricted CTL HBV envelope epitope peptide (WLSLLVPFV) as an unrelated peptide control. The epitope peptide mixture (mix1∼mix14) contains 2 to 6 single epitopes in each mixture. **A**. GrB SFCs induced by single peptide in self-limited and chronic HCV infection. **B**. GrB SFCs induced by multiple epitope peptide mixtures in self-limited and chronic HCV infection; *P<0.05.

The cytotoxic function of CTLs plays an important role in eliminating the hepatitis C virus from the host. To detect whether epitope-specific cytotoxic T cell responses were induced by mDCs loaded with single peptide or multiple-epitope peptide mixtures, we used GrB ELISPOT assay (similar to a ^51^Cr-release assay) to monitor T cell-mediated cytotoxicity [32], [33]. Interestingly, we found that single-peptide pulsed DCs could significantly enhance the number of GrB SFCs in the self-limited HCV infection samples compared to no peptide control, but not in the chronic HCV infection samples (Figure 3A). However, we found that multiple-epitope peptide mixtures pulsed DCs not only increased the number of GrB SFCs in the self-limited HCV infection samples, but also in chronic HCV infection samples. Especially three multiple-epitope peptide pools (mix2,mix6 and mix7) could induce the more number of GrB SFCs in chronic HCV infection than in self-limited HCV infection and there was a significant difference in mix6 (P<0.05) between self-limited infection and chronic HCV infection (Figure 3B). We also observed that the epitope peptide mixtures that induced the high number of GrB SFCs included the same novel immunodominant epitope peptides, which were NS2 (834–842) and NS4a (1700–1708). Importantly, this CTL immune response was HCV-epitope specific, because HLA-A2 restricted CTL epitope from HBV didn’t induce appreciable GrB SFCs (Figure 3A–B). Taken together, our results suggest that multiple-epitope peptide mixtures of immunodominant peptides that are presented on mDCs can induce a cytotoxic response from both self-limited HCV and chronic HCV infection patient CTLs in an antigen-specific manner.

### Th1-biased Immune Response was Induced through DCs Pulsed with Immunodominant Epitope Peptides

**Figure 4:**
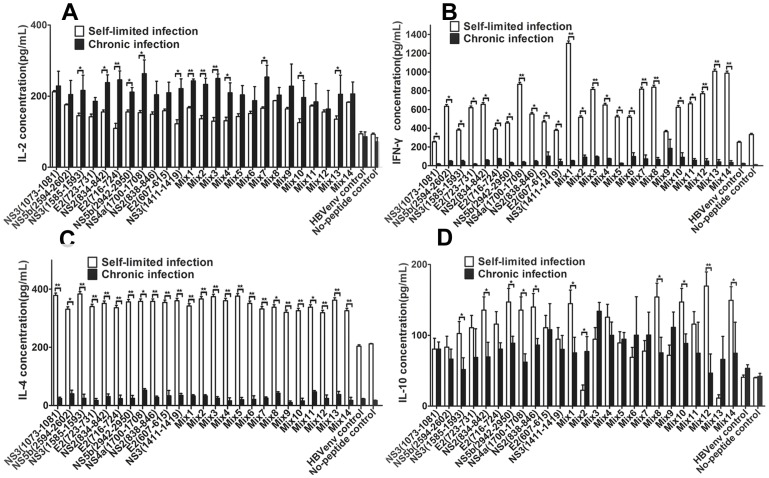
Cytokine concentrations in supernatants from T lymphocytes cocultured with DCs(pg/mL). The data was expressed as mean±SEM, IL-2, IFN-γ, IL-4, and IL-10 concentration in the supernatants from T lymphocytes in 4 self-limited HCV infections and 5 chronic HCV subjects, cocultured with DCs loaded with single epitope peptide or multiple epitope peptide mixtures. Triplicate wells of every peptide in the same experiment were used. The single peptides include 4 previously reported epitope peptides (E2 (723–731), NS3 (1073–1081), NS3 (1585–1593), and NS5b (2594–2602)), 7 predicted peptides and HLA-A2 restricted CTL HBV envelope epitope peptide (WLSLLVPFV) as an unrelated-peptide control. The epitope peptide mixture (mix1∼mix14) contains 2 to 6 single epitopes in each mixture. **A**. IL-2 concentration. **B**. IFN-γ concentration. **C**. IL-4 concentration. **D**. IL-10 concentration. *P<0.05, **P<0.01.

The cytokine spectrums from the responder T cells co-cultured with mDCs pulsed with single peptide or multi-epitope peptide were detected from the supernatant harvested from the same cells used for the cytotoxicity assay. While high levels of both IFN-γ and IL-4 were observed in self-limited HCV infection (Figure 4B, 4C), and we confirmed high levels of IL-2 and IL-10 in chronic HCV infection (Figure 4A, 4D). There was a significant difference between self-limited HCV infection and chronic HCV infection subjects (Figure 4A–D). Overall, our data collectively suggests that not only single peptides but also multiple-epitope peptide mixtures could induce a Th1-biased immune response, although a complicated cytokine spectrum was detected in the study subjects. These results are meaningful in light of the differences in protective immune response against hepatitis C infection between the chronic HCV and self-limited subjects.

## Discussion

Although the mechanism of hepatitis C infection persistence is complex, a strong, broad, and multiple-epitope T cell response is associated with the termination of hepatitis C [11]–[13], [34]. Therefore, the selection of immunodominant CTL epitopes may play an important role when developing therapeutic strategies to induce specific T cell responses, especially via epitope peptide-pulsed DCs [20], [22]. In this study, we screened the HCV proteome and identified 7 novel HLA-A2-restricted HCV-specific CTL epitopes in the Chinese population that demonstrated a strong stimulating ability to induce epitope-specific IFN-γ secretion, especially in self-limited infections. Furthermore, mature DCs pulsed with immunodominant epitopes could not only significantly increase IFN-γ secretion and GrB expression in T lymphocytes, but also induce a Th1-biased cytokine immune response. Overall, the results from this study indicate that these peptides could induce a protective immune response and could therefore serve as candidate peptides for the development of immunotherapy for chronic HCV infection.

In recent years, many HCV immunodominant epitope peptides have been identified in distinct regions throughout the HCV proteome through different methods of identification [6], [35]–[37]. However, of note, inappropriate epitope peptides might induce an immune response that is not conducive to eliminate the HCV infection [38]. Moreover, the synthesis of a peptide pool containing all possible peptides would be costly. Therefore, we sought out in this study to identify a few immunodominant epitopes that have both high sensitivity and specificity by using a bioinformatics approach using the online CTLpred software tool followed by a functional IFN-γ ELISPOT assay. Each of the 7 peptides identified by this process had a strong stimulating capacity for IFN-γ secretion and had relative conservation in HCV genotype 1 and 2 (data not shown). This conservation in other immunodominant epitope peptides has also been found in subjects that recovered from HCV infection [39], [40]. In addition to revealing novel peptides by this screening method, we also selected 9 previously reported HLA-A2-restricted hepatitis C virus CTL epitopes by using the text mining; these already established peptides have been shown to induce a strong effect T cells response in both self-limited HCV infection and chronic infection. Surprisingly, only 4 peptides were finally identified as a positive epitopes according to our criteria (Table 2). This phenomenon indicates that immunodominant epitope peptides might relate to the different patient populations. However, this needs further investigation.

DCs have potent functions in the immune response, including capturing and processing antigens, migrating to lymph nodes, expressing co-stimulating molecules, and producing cytokines that activate T lymphocytes. Although some studies have reported impaired DC function in chronic hepatitis C infected patients [6]–[8], we found in this study that there was no significant difference in co-stimulation molecule expression between chronic HCV infection and self-limited HCV subjects when we used a cytokine cocktail and toll-like receptor ligand (PolyI:C) to promote monocyte-derived DC maturation (Figure S1). Furthermore, the mature DCs secreted higher levels of IL-12 than IL-10(data not shown). This finding is consistent with past studies [31], [41]. Taken together, these characteristics suggested that mature DCs could switch on a Th1-biased immune response and promote T cell responses for immunotherapeutic purposes, as the inhibition of DC function is associated with promoting CD8^+^ T cell exhaustion during chronic HCV infection [42].

Some evidence has indicated that multiple epitope peptides from different regions of the HCV genome are necessary to generate strong immunogenicity and stimulate a wide range of epitope-specific CD8^+^ T cell responses [43]. Moreover, effective induction of the protective immune response depends on the appropriate combination of several epitope peptides within the set of peptides to use for immunotherapy [44]. We therefore selected 6 novel peptides identified in this study and 3 previously reported HLA-A2-restricted hepatitis C virus CTL epitopes to comprise the multiple-epitope peptide mixtures, as these peptides had a stronger stimulatory function as compared to the others we functionally tested. Before mDCs pulsed with these multiple-epitope peptide mixtures, we found that these epitope peptide mixtures could induce more IFN-γ production than IL-4 from PBMCs harvested from chronic HCV infected subjects (data not shown). Then we observed that mDCs pulsed with single peptide or multiple-epitope peptide mixtures could induce significantly more multi-epitope specific T cell responses than the controls, although different antigen-recognition profiles were detected from different subjects. These pulsed mDCs mainly induced high number of IFN-γ secretion in self-limited HCV infection and chronic HCV infection, especially multiple-epitope peptide mixtures in chronic HCV infection (Figure 2B). However, as we expected, we found that only multiple-epitope peptide mixtures, not single epitopes, could increase higher number of GrB secretion of CD8^+^ T cells in chronic hepatitis C infection than in self-limited HCV infection (Figure 3). These results demonstrate that multiple epitope peptide mixtures can induce a broad T cell response and these T cell response might come from the clone expansion of memory T cells [6], [11].

Previous work found that peptides from non-structural proteins were able to stimulate CTL responses and that multiple epitope peptide sequences were recognized by T cells within these non-structural regions [45]. We further investigated whether our multi-epitope peptide mixtures contain these same peptides; indeed, we found that some of the peptides we identified were distributed in non-structural protein regions of HCV. They were NS2 (834–842), NS4a (1700–1708), and NS5b (2942–2950). However, we also investigated multi-epitope peptide mixtures that contain more than 5 peptides and found that they were not able to significantly increase IFN-γ or GrB secretion in the study subjects. This suggests that just having more peptides in an epitope peptide mixture may not necessarily translate into a better epitope peptide mixture in terms of functional capacity to induce T cell effect function. The mechanism behind what constitutes an appropriate and effective stimulatory epitope peptide mixture will be explored in future studies.

In addition to the cytotoxic role that CD8^+^ T lymphocytes play in the adaptive immune response during pathogen infection, they also simultaneously secrete some cytokines, such as IL-2, IFN-γ, and TNF-α. These cytokines play a crucial role in eliminating foreign pathogens [10], [34], [46]. In this study, we found that single peptide or multiple-epitope peptide mixtures could induce the production of high levels of Th1-biased immune response cytokines (Figure 4A–D), although a different cytokine spectrum was found when comparing self-limited HCV infection to chronic HCV infection. In contrast, many studies have shown that a Th2-biased cytokine immune response is present in persistent/chronic HCV infection [47]–[49]. Although PBMCs stimulated by mDC pulsed with 9-mer peptides, it is worth noting that cytokines might also be secreted by CD4^+^T cells, because cross-reactivity T cells exist during the chronic infection. The mechanism needs to be investigated in the future. Within our data, the cytokine profile we found was at least consistent with the IFN-γ and GrB SFCs results, for example, there were high levels of IFN-γ in coculture supernatants and low IFN-γ SFCs in the self-limited HCV infection; However, this was converse in chronic HCV infection(Figure 2 and Figure 4B). This indicated that the memory T cells in self-limited HCV infection subjects can quickly clonal expansion when faced with hepatitis C virus antigen again. However, as persisting HCV infection or anergy of T cells, inducing of the immune response in chronic HCV infection was slow. These results further suggest that DCs pulsed with novel multiple-epitope peptide mixtures can induce a wide range of multi-epitope–specific T lymphocyte responses.

As described above, we identified 7 novel HLA-A2-restricted HCV-specific CTL epitope peptides, and these peptides can significantly increase the epitope-specific T cell response in self-limited and chronic HCV infection subjects. But the number of study subjects in this study is small; to confirm and extend these results, we need to enlarge the study population in future studies. Furthermore, this study only focused on the highly prevalent HLA-A2-restricted epitopes in the population. However, other HLA-I/II alleles have also been shown to be associated with spontaneous clearance of hepatitis C infection [40], [50], [51], and these would be interesting to study in the future as well. Additionally, the detailed mechanism behind the different cytokine spectrum observed in different subjects needs to be examined closely in future studies. Overall, As DCs are characterized by potent immunostimulatory properties in activating and attracting T cells. And based on previous research in HIV infection and in tumor immunotherapy, DCs pulsed with immunodominant epitope peptides as an alternative strategy would be more efficient, safer, and more feasible for hepatitis C immunotherapy than currently available treatments [18], [19], [20], [23], [52], especially in refractory hepatitis C virus.

In conclusion, we screened and identified seven novel HLA-A2 restricted hepatitis C virus specific CTL epitope peptides through predictive screening by bioinformatics and functional testing by IFN-γ ELISPOT. We also found that DCs pulsed with multiple-epitope peptide mixtures could increase the IFN-γ and GrB secretion of HCV-epitope-specific T cells above that of the unrelated peptide and negative control. Moreover, the stimulation induced by these peptides could induce a Th1-biased T cell immune response, suggesting that these peptides could induce epitope-specific T cells against HCV infection. Therefore, this study not only reveals a promising immunodominant epitope peptide candidate for designing effective therapeutic vaccines, but also provides evidence that DC-based immunotherapy could work to help eliminate HCV virus from chronic HCV-infected subjects.

## Supporting Information

Figure S1 **Surface molecules of monocyte-derived mature DC (%).** The data was expressed as mean±SEM; data from 3 chronic HCV infection and 3 self-limited HCV infection subjects was included. “ns” means no significance between self-limited HCV infection and chronic HCV infection.
(TIF)

Table S1 **Epitope peptide mixtures from mix1 to mix14.** Note: The epitope peptide mixtures include six novel epitopes and three past reported epitopes, these epitope peptides had a strong stimulating function of IFN-γ secretion in self-limited infection.
(DOC)
