# Correction: DCs Pulsed with Novel HLA-A2-Restricted CTL Epitopes against Hepatitis C Virus Induced a Broadly Reactive Anti-HCV-Specific T Lymphocyte Response

**DOI:** 10.1371/annotation/2ea71143-c033-43ff-8282-781b76c0a054

**Published:** 2012-09-17

**Authors:** Zhongsheng Guo, Henghui Zhang, Huiying Rao, Dong Jiang, Xu Cong, Bo Feng, Jianghua Wang, Lai Wei, Hongsong Chen

There was an error in affiliation 1. Affiliation 1 should be: Peking University Hepatology Institute, Peking University People’s Hospital, Beijing, China

