# Supplementary figures and images for: DCs Pulsed with Novel HLA-A2-Restricted CTL Epitopes against Hepatitis C Virus Induced a Broadly Reactive Anti-HCV-Specific T Lymphocyte Response
Source: PLoS One. 2012 Jun 12;7(6):e38390. doi: 10.1371/journal.pone.0038390 (PMC3373515; doi:10.1371/journal.pone.0038390)

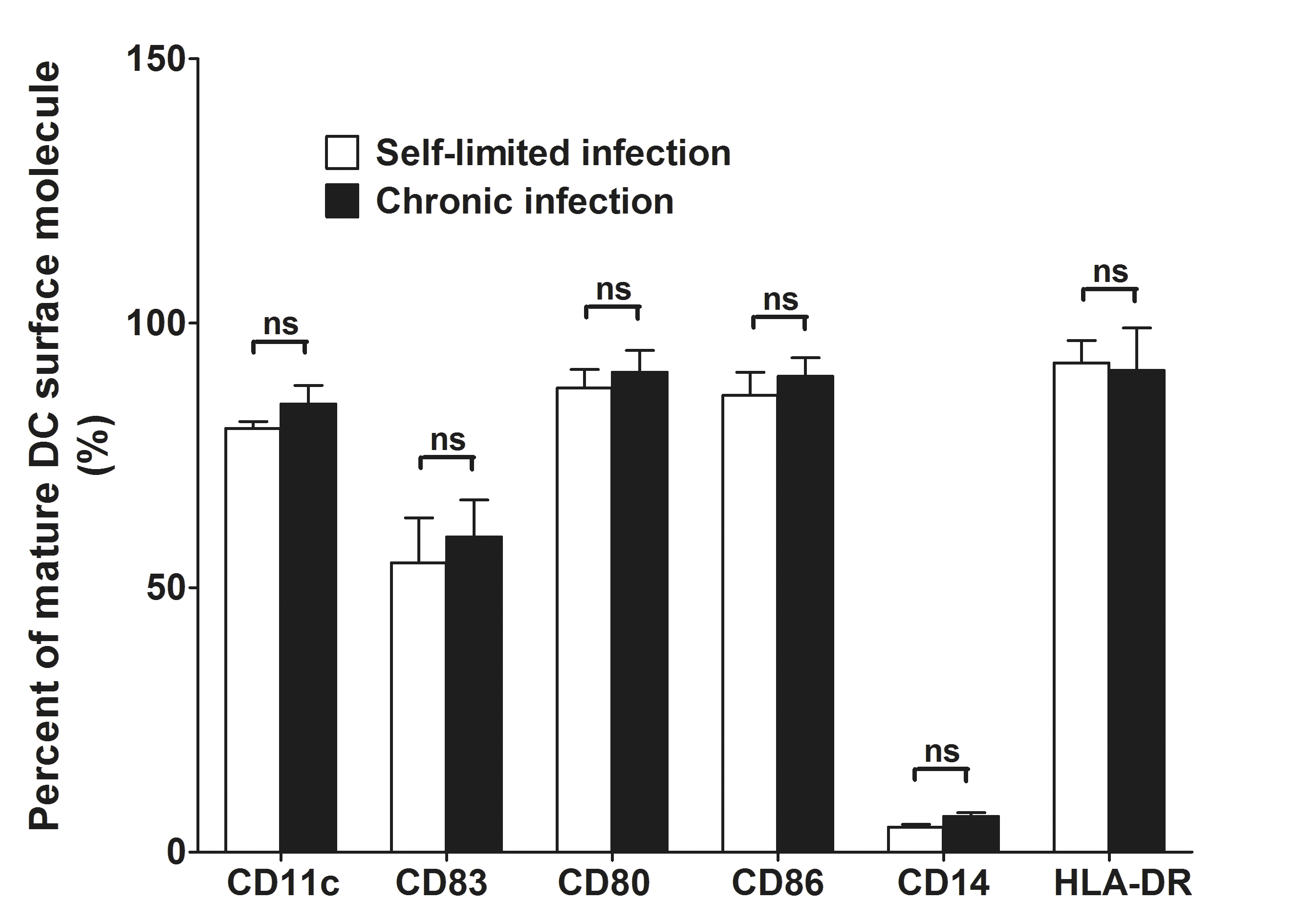

Supplement: Figure S1 — Surface molecules of monocyte-derived mature DC (%). The data was expressed as mean±SEM; data from 3 chronic HCV infection and 3 self-limited HCV infection subjects was included. “ns” means no significance between self-limited HCV infection and chronic HCV infection. (TIF) [file pone.0038390.s001.tif]
