# Supplementary material for: DCs Pulsed with Novel HLA-A2-Restricted CTL Epitopes against Hepatitis C Virus Induced a Broadly Reactive Anti-HCV-Specific T Lymphocyte Response
Source: PLoS One. 2012 Jun 12;7(6):e38390. doi: 10.1371/journal.pone.0038390 (PMC3373515; doi:10.1371/journal.pone.0038390)
Supplement: Table S1 — Epitope peptide mixtures from mix1 to mix14. Note: The epitope peptide mixtures include six novel epitopes and three past reported epitopes, these epitope peptides had a strong stimulating function of IFN-γ secretion in self-limited infection. (DOC) [file pone.0038390.s002.doc]

**Table S1. Epitope peptide mixtures from mix1 to mix14**

| **Epitope mixture** | **Composition of epitope peptide mixtures** |
| --- | --- |
| **Mix1** | E2(716-724),E2(723-731) |
| **Mix2** | NS2(834-842),NS3(1073-1081),NS4a(1700-1708),NS5b(2942-2950) |
| **Mix3** | E2(723-731),NS2(834-842), NS4a(1700-1708), NS5b(2942-2950) |
| **Mix4** | E2(716-724),NS3(1073-1081),NS4a(1700-1708),NS5b(2594-2602) |
| **Mix5** | E2(716-724),NS5b(2594-2602),NS5b(2942-2950) |
| **Mix6** | NS2(834-842),NS4a(1700-1708),NS5b(2594-2602),NS5b(2942-2950) |
| **Mix7** | E2(723-731),NS2(834-842),NS3(1073-1081),NS4a(1700-1708),NS5b(2594-2602) |
| **Mix8** | E2(723-731),NS2(834-842),NS3(1073-1081),NS4a(1700-1708),NS5b(2942-2950) |
| **Mix9** | E2(716-724),NS2(834-842),NS3(1073-1081),NS4a(1700-1708),NS5b(2594-2602) |
| **Mix10** | E2(716-724),NS2(834-842),NS3(1073-1081),NS4a(1700-1708),NS5b(2942-2950) |
| **Mix11** | E2(716-724),E2(723-731),NS2(834-842),NS3(1073-1081),NS4a(1700-1708),NS5b(2594-2602) |
| **Mix12** | E2(716-724),E2(723-731),NS2(834-842),NS3(1073-1081),NS4a(1700-1708),NS5b(2942-2950) |
| **Mix13** | E2(723-731),NS2(834-842),NS3(1073-1081),NS4a(1700-1708),NS5b(2594-2602),NS5b(2942-2950) |
| **Mix14** | E2(716-724),NS2(834-842),NS3(1073-1081),NS4a(1700-1708),NS5b(2594-2602),NS5b(2942-2950) |
